# Bee Pollen and Probiotics’ Potential to Protect and Treat Intestinal Permeability in Propionic Acid-Induced Rodent Model of Autism

**DOI:** 10.3390/metabo13040548

**Published:** 2023-04-12

**Authors:** Mona Alonazi, Abir Ben Bacha, Mona G. Alharbi, Arwa Ishaq A. Khayyat, Laila AL-Ayadhi, Afaf El-Ansary

**Affiliations:** 1 Department of Biochemistry, College of Science, King Saud University, P.O. Box 22452, Riyadh 11495, Saudi Arabia; 2 Department of Physiology, Faculty of Medicine, King Saud University, P.O. Box 2925, Riyadh 11461, Saudi Arabia; 3 Central Laboratory, King Saud University, P.O. Box 22452, Riyadh 11495, Saudi Arabia

**Keywords:** autism spectrum disorder, leaky gut, oxidative stress, bee pollen, probiotics

## Abstract

Rodent models may help investigations on the possible link between autism spectrum disorder (ASD) and gut microbiota since autistic patients frequently manifested gastrointestinal troubles as co-morbidities. Thirty young male rats were divided into five groups: Group 1 serves as control; Group 2, bee pollen and probiotic-treated; and Group 3, propionic acid (PPA)-induced rodent model of autism; Group 4 and Group 5, the protective and therapeutic groups were given bee pollen and probiotic combination treatment either before or after the neurotoxic dose of PPA, respectively. Serum occludin, zonulin, lipid peroxides (MDA), glutathione (GSH), glutathione-S-transferase (GST), glutathione peroxidase (GPX), catalase, and gut microbial composition were assessed in all investigated groups. Recorded data clearly indicated the marked elevation in serum occludin (1.23 ± 0.15 ng/mL) and zonulin (1.91 ± 0.13 ng/mL) levels as potent biomarkers of leaky gut in the PPA- treated rats while both were normalized to bee pollen/probiotic-treated rats. Similarly, the high significant decrease in catalase (3.55 ± 0.34 U/dL), GSH (39.68 ± 3.72 µg/mL), GST (29.85 ± 2.18 U/mL), and GPX (13.39 ± 1.54 U/mL) concomitant with a highly significant increase in MDA (3.41 ± 0.12 µmoles/mL) as a marker of oxidative stress was also observed in PPA-treated animals. Interestingly, combined bee pollen/probiotic treatments demonstrated remarkable amelioration of the five studied oxidative stress variables as well as the fecal microbial composition. Overall, our findings demonstrated a new approach to the beneficial use of bee pollen and probiotic combination as a therapeutic intervention strategy to relieve neurotoxic effects of PPA, a short-chain fatty acid linked to the pathoetiology of autism.

## 1. Introduction

Autism spectrum disorder (ASD) has been linked to gastrointestinal (GI) issues. Nearly half of the children with ASD have at least one GI symptom [1] and they are more likely to experience GI symptoms than their neurotypical peers [2], with diarrhea and constipation being the most often reported symptoms [3]. Furthermore, new research indicates a substantial correlation between the severity of GI symptoms and the severity of autistic symptoms [4,5,6]. These results suggest that the gut environment may play a crucial role in the etiology of ASD.

The bidirectional link between the brain and the GI tract (the gut–brain axis) makes the existence of elevated blood metabolites in ASD important [7]. Through the neuroimmune, neuroendocrine, and autonomic nervous systems, the “leaky gut” influences brain function and may have a role in the development of autistic features [8,9]. It follows that the altered metabolites found in the urine and systemic circulation of individuals with ASD may have an impact on the brain and neurodevelopment.

Lactulose/mannitol ratio studies of intestinal permeability in ASD patients revealed higher permeability compared to healthy controls [10]. However, the mechanism of increased intestinal permeability in people with ASD is yet unknown. Many investigations have found that occludin and zonulin may be biomarkers of epithelial barrier permeability [11].

Occludin is an important tight junction (TJ) protein known to be associated with epithelial permeability [12]. Occludin contributes to the stability and integrity of TJs, thus regulating and restricting the paracellular transport pathway [13]. Occludin mutant or knockout animals exhibited chronic inflammation and a defective epithelial barrier despite having morphologically intact TJs; this demonstrated that it plays a critical role in maintaining barrier stability rather than TJ assembly [14,15].

Li et al. [16] investigated the protective effects of the bee pollen extract on intestinal barrier function and identified its anti-inflammatory and antioxidant impacts on the regulation of key cytokine gene expression. Additionally, they discovered that the pretreatment of Caco-2 cells with bee pollen extract significantly decreased the activation of the MAPK signaling pathway in response to the dextran sulfate sodium (DSS)-induced cytotoxic damage to intestinal cells. Surprisingly, the pretreatment bee pollen-Cs extract group was still able to sustain cellular metabolism equal to the blank group untreated with DSS [16].

Antioxidant qualities and activities are thought to protect against the damaging effects of highly reactive oxygen species (ROS) and thereby treat oxidative stress-related illnesses. Several chronic diseases, as well as disease progression, can be prevented and slowed by strengthening the body’s antioxidant defenses through the consumption of antioxidant-rich foods or dietary supplements [17]. The product of enteric fatty acid bacterial fermentation known as propionic acid (PPA) can have significant impacts on the stomach, brain, and behavior. Numerous neurochemical abnormalities are present in the brain tissue of PPA-treated rats, including neuroinflammation, glutamate excitotoxicity, oxidative stress, GSH depletion, and altered membrane phospholipids, which are found in ASD patients. PPA also possesses other bioactive qualities that affect the immunological response, intestinal permeability, mitochondrial function, and neurotransmitter systems. The use of PPA in rats as a reliable animal model of ASD is supported by the fact that all of these PPA-induced changes are congruent with the symptoms and the main hypothesized etiological pathways of ASD [18,19]. Using PPA as a food preservative and additive to colorful food (such as in canned foods, dairy products, Brazilian bread, etc.), makes PPA a hot issue for research [19].

Based on the interesting gut-protective properties of bee pollen and its potential regulatory effects on glycerophospholipid and sphingolipid metabolisms, which may be required for constructing permeability barriers and reducing intestine oxidative stress [20], as well as the potential of probiotic strains in controlling the expression of TJ-specific genes and proteins required for proper intestinal barrier’s function and integrity [21], the current work aimed to measure serum occludin and zonulin as markers of leaky gut and impaired gut microbiota, as well as selected variables related to oxidative stress in PPA-induced rodent models compared to control healthy untreated rats, and combined bee pollen and probiotic protected or therapeutically treated animals. This could help to demonstrate the benefits of symbiotic antioxidant capabilities and action pathways in alleviating increased intestinal permeability as neurotoxic effects of PPA in a rodent model of ASD related to gastrointestinal comorbidity in individuals with ASD.

## 2. Materials and Methods

### 2.1. Animal Experiments

Animal experiments were performed on thirty male Wister albino rats (3 weeks old, ~80 g) which were housed in groups in cages (26.5 cm × 14.5 cm × 42.5 cm) under controlled laboratory conditions (temperature 23 °C, humidity 55 ± 5% and day/night 12 h light cycle). All animals had free access to standard diet (AIN-93 G, Grain Silos and Flour Mills organization, Riyadh, Saudi Arabia). Every three rats were placed in a separate cage for 7 days to become acclimatized, before being randomly divided into five groups of six rats each as follows: (1) control group getting only phosphate buffered saline; (2) bee pollen and probiotics group was orally administered with a combination of bee pollen (NZ Bee Pollen Granules, Happy Valley, New Zealand) and probiotics (PROTEXIN^®^, Somerset, UK), a mixture of some healthy bacteria including *Bifidobacterium breve*, *Bifidobacterium infantis*, *Lactobacillus acidophilus*, *Lactobacillus bulgaricus*, *Lacticaseibacillus casei*, *Lacticaseibacillus rhamnosus*, and *Streptococcus thermophiles*. Probiotics International Limited, Somerset, UK) meeting the dose of 200 mg/kg body weight [22]; (3) PPA group received PPA (Sigma-Aldrich, Burlington, MA, USA) meeting dose of 250 mg/kg body weight for last consecutive three days [23]; (4) therapeutic group was administered with the same dose of PPA followed by bee pollen and probiotics combination at the dose 200 mg/kg body weight; (5) protective group receiving, at the same doses, bee pollen and probiotics combination orally followed by PPA. The bee pollen granules were first weighed and ground then dissolved in distilled water with probiotics and orally administered to animals with oral gavage.

On day 28, blood sample was collected in a plain tube without anticoagulant by direct cardiac puncture. Serum samples were obtained after centrifugation at 3000 rpm, 4 °C for 10 min, and were stored at −80 °C until use. The experimental procedure was pre-approved by the ethics committee for animal research of King Saud University, Riyadh (ethics reference number: KSU-SE-19-35).

### 2.2. Biochemical Analyses

Methods described by Ruiz-Larrea et al. [24] and Beutler et al. [25] were used to measure lipid oxidation by the formation of thiobarbituric acid reactive substances and glutathione (GSH) level by using 5,5′-dithiobis 2-nitrobenzoic acid and sulfhydryl compounds, respectively.

The activities of catalase and glutathione-S-transferase (GST) were investigated according to the methods of Maehly and Chance [26] and Mannervik [27], respectively, by following the rate of hydrogen peroxide dissociation/minute by the catalase enzyme or the rate of formation of dinitrophenyl thioether/minute by the GST enzyme which can be detected by spectrophotometer at 240 nm or 340 nm, respectively. Likewise, glutathione peroxidase (GPX) activity was assayed according to the method of Paglia and Valentine [28] by monitoring the change in absorbance at 340 nm due to NADPH oxidation.

Serum occludin and zonulin in all groups were investigated using ELISA kits, products of MyBioSource following the manufacturers’ instructions. All measurements were performed in triplicate, and the mean of three different readings was calculated. Quality control assays were performed to evaluate experimental reproducibility through the inter- and intra-assay coefficients of variability (%CV).

### 2.3. Microbial Analysis

The fecal specimens were weekly collected in the morning from all groups in sterile tubes and stored at −80 °C. Later, the frozen tubes were homogenized for 5 s in a vortex mixer and centrifuged (4000 rpm, 3 min, −4 °C). The obtained fecal suspensions were first subjected to tenfold serial dilutions with PBS solution (dilution 1). Then the process was repeated until reaching dilution 4. An amount of 100 µL of each of the resulting dilutions was smeared on the surface of various culture media: MacConkey agar (MCA) without crystal violet dye for distinguishing *Enterobacteriacea* (Gram-negative rod, lactose fermenters [29], nutrient agar (NA) for the distinguishing *Bacilli* (Gram-positive or negative rod) after thermic treatment at 80 °C for 10 min [30], sulfite polymyxin sulfadiazine (SPS) agar to distinguish *Clostridium botulinum* [31], Sabouraud’s dextrose agar to identify *Candida albicans* [32] and Mueller–Hinton agar (MHA) to identify *Moraxella* spp. (Gram-negative) [33], blood agar for distinguishing Gram-positive/negative rod and cocci. MHA, MCA NA, and blood agar plates [33] were incubated under aerobic conditions for 24 h at 37 °C while SPS agar plates were incubated anaerobically for 24–48 h at 35 ± 2 °C [34]. Colony morphology based on size, color, and shape was evaluated microscopically on the microscope slide through the Gram staining method. White smooth colonies of *C. albicans* on Sabouraud’s dextrose agar were examined and confirmed under microscope [32].

The data from the culture-based methods were quantified based on a ++++ scale defined as colony-forming unit (CFU), a measure of viable bacterial or fungal numbers. The number of CFU on the plates was counted for each dilution. (+) = Rare, less than 10^3^ CFU/g of feces; (++) = Few, 10^3^–10^4^ CFU/g of feces; (+++) = Moderate, 10^5^–10^6^ CFU/g of feces; (++++) = Heavy, >10^6^ CFU/g of feces.

Then, every well-isolated colony, regardless of appearance, was picked in succession (top of the plate). Each isolate was characterized according to Holdeman et al. [35] procedure. Briefly, the colonies were spread on the slide. Smears were heat-fixed, gently Gram-stained, and then examined under a microscope using oil immersion lens.

It is worth noting that traditional microbial identification processes are only partially selective and serve only as a starting step in the identification of microorganisms. Therefore, in further work, it is necessary to use additional methods to determine which microbes are actually present in the fecal samples. The determination of the level of genera/species will be further improved in further work by selecting additional selective microbiological media, as well as by introducing biochemical tests and molecular characterization.

### 2.4. Statistical Analysis

All data were carried out by *one-way ANOVA* followed by Tukey’s multiple comparison test. Only *p* values ≤ 0.05 were considered significant. Results illustrated as mean ± standard error of the mean (SEM) were obtained using GraphPad prism (version 9.5.0).

## 3. Results

**Figure 1:**
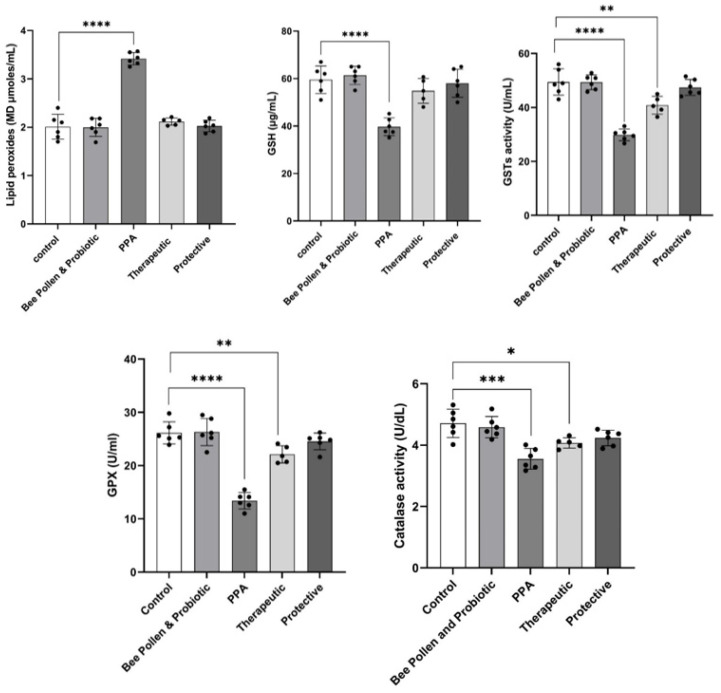
Effect of PPA treatment, protective, and therapeutic effects of bee pollen/probiotics on serum oxidative stress-related variables. Data presented are means ± standard error. Multiple comparisons by Tukey’s post hoc test * *p*  ≤  0.050, ** *p*  ≤  0.01, *** *p*  ≤  0.001, **** *p*  ≤  0.0001. GSH, glutathione; GPX, glutathione peroxidase, GST, glutathione-S-transferase; PPA, propionic acid.

Figure 1 demonstrates the highly significant increase in MDA (3.41 ± 0.12 µmoles/mL) as a marker of oxidative stress (*p*  ≤  0.0001) concomitant with a highly significant decrease in GSH (39.68 ± 3.72 µg/mL), GST (29.85 ± 2.18 U/mL), and GPX (13.39 ± 1.54 U/mL) (*p*  ≤  0.0001) in PPA-treated group as a rodent model of ASD. A significant reduction of catalase (3.55 ± 0.34 U/dL) in PPA-treated animals was also observed (*p*  ≤  0.001). On the other hand, bee pollen/probiotic treatments demonstrate remarkable amelioration of the five studied oxidative stress variables. While the therapeutically treated group of rats still show less significant altered lower levels of GSH (54.80 ± 5.23 µg/mL), GST (40.84 ± 3.30 U/mL), GPX (22.11 ± 1.62 U/mL), and catalase (4.07 ± 0.17 U/dL), it recorded nonsignificant levels of MDA (2.12 ± 0.07 µmoles/mL) as a marker of oxidative stress compared to controls healthy rats (59.5 ± 5.8 µg/mL; 49.42 ± 4.86 U/mL, 26.15 ± 2.08 U mL, 4.71 ± 0.46 U/dL and 2.01 ± 0.02 µmoles/mL, respectively). Figure 1 also presents that the protective group has more or less the same levels of the five oxidative stress measured variables (MDA (2.02 ± 0.12 µmoles/mL; GSH: 58 ± 6 µg/mL; GST 47.45 ± 2.95 U/mL; GPX: 24.53 ± 1.56 U/mL; catalase: 4.23 ± 0.25 U/dL)).

**Figure 2:**
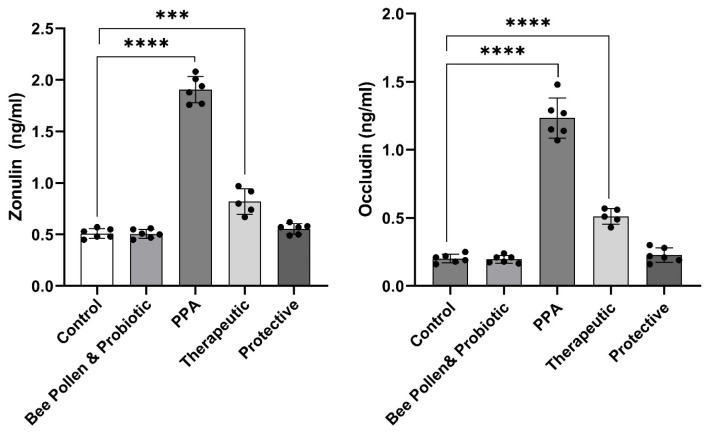
Effect of PPA treatment, protective, and therapeutic effects of bee pollen/probiotics on serum intestinal permeability related variables. Data presented are means ± standard error. Multiple comparisons by Tukey’s post hoc test *** *p*  ≤  0.001, **** *p*  ≤  0.0001. PPA, propionic acid.

Figure 2 demonstrates the same trend in relation to intestinal permeability measured biomarkers (*p*  ≤  0.0001). While zonulin and occludin were significantly higher in PPA-treated rats (1.91 ± 0.13 ng/mL and 1.23 ± 0.15 ng/mL, respectively), compared to the control group (0.51 ± 0.05 ng/mL and 0.20 ± 0.03 ng/mL, respectively), showing gut leakiness, both proteins were normalized in bee pollen/probiotic-treated rats. Indeed, recorded data demonstrated zonulin levels of 0.82 ± 0.12 ng/mL or 0.55 ± 0.05 ng/mL and occludin levels of 0.51 ± 0.06 ng/mL or 0.23 ± 0.05 ng/mL in therapeutic or protected groups, respectively.

Table 1 demonstrates changes in bacteria growth in the feces of treated rats compared to the control. The most important noticed variation is the disappearance of *Candida albicans* in the protected groups along the experimental duration together with the disappearance of *Clostridium botulinum* on the 3rd and 4th week of our experiment

## 4. Discussion

The analysis of the results presented in Figure 1 and Figure 2, in which the synergistic therapeutic and protective effects of bee pollen and probiotic on selected oxidative stress variables and zonulin and occludin as biomarkers of intestinal permeability, experimentally induced post-oral administration of a neurotoxic dose of PPA, showed that the combination exhibited strong antioxidant effects presented as lower lipid peroxides concomitant with a remarkable increase in GSH, GST, GPX, and catalase activities, and anti-gut leakiness effects presented as significantly lower serum zonulin and occludin as markers of increased intestinal permeability (Figure 1).

Many studies and analyses on autistic children have found that blood samples from individuals with ASD have higher levels of oxidative stress indicators such as oxidized glutathione (GSSG), S-adenosyl homocysteine, copper homocysteine, and malondialdehyde than healthy control groups [36,37,38,39]. As opposed to healthy control children, autistic children’s levels of total GSH (tGSH), blood-reduced glutathione (GSH), GSH/GSSG, tGSH/GSSG, methionine, cysteine, S-adenosyl methionine/S-adenosyl homocysteine, and calcium were noticeably lower. All of these findings and information point to oxidative stress markers as the primary indicator of the pathophysiology of autism [36]. An oxidative stress marker in brain homogenate revealed increased lipid peroxidation, protein carbonyl, and GST activity, along with a decrease in GSH and GPX activity, in a model of autism treated with PPA, according to previous research [36]. Therefore, earlier studies supported the PPA-induced oxidative stress in our present study [37,38,39].

Figure 1 also demonstrates the synergistic therapeutic and protective antioxidant effects of bee pollen and probiotic supplementation in the PPA-induced rodent model of ASD. These effects can be noticed as a remarkable reduction of MDA levels concomitant with a remarkable increase in GSH, GPX, GST, and catalase as antioxidants. This can find support in previous studies, which prove the antioxidant effects of both products (bee pollen and probiotics) [40,41]. *Lactobacillus* strains are known to be resistant to many forms of reactive oxygen species (ROS), including superoxide anions, peroxide radicals, and hydroxyl radicals, according to the data [42,43,44,45]. In addition to their anti-inflammatory and antiapoptotic effects, probiotics have been shown to have antioxidant potential in studies conducted recently [41,46,47]. For instance, the probiotic *Bifidobacterium animalis* reduces free radicals in vitro and boosts antioxidant activity in mice [47]. Moreover, *Lacticaseibacillus rhamnosus GG* (LGG) has been shown to have potential antioxidant properties [44].

The antioxidant effects of the used mixture of probiotics can be attributed to their regulatory effects of the NADPH oxidase (NOX) complex as a major source of ROS. According to studies, the probiotics *Limosilactobacillus fermentum* CECT5716, *Lactobacillus coryniformis* CECT5711 (K8), and *Lactobacillus gasseri* CECT5714 (LC9) reduce NOX activity and decrease the mRNA expression of NOX-1 and NOX-4 enzymes, as a result, they reduce the level of ROS production. Close support to our data is the fact that probiotics also increase antioxidant activity within the host by raising the concentration and activity of a number of enzymes. Studies have shown, for instance, that the probiotic *Limosilactobacillus fermentum* and *Bifidobacterium lactis Bb12* raise levels of superoxide dismutase (SOD), GPX, catalase, and Cu, Zn-SOD enzymes either clinically or experimentally [48,49].

Phenolic compounds, including flavonoids and phenolic acids, are recognized as important natural antioxidants, as well as molecules with anti-inflammatory, antioxidant, antibacterial, antiallergic, antiviral, antithrombotic, hepatoprotective, and signaling properties [50]. Consumption of phenolic compounds has been linked to a lower risk of developing chronic diseases, as evidenced by epidemiological research [51,52].

The significant synergistic benefits of combined probiotics and bee pollen could also be attributed to the principal antioxidant components of bee pollen, among which are phenolic acids and flavonoids, which are plant-derived polyphenolic chemicals [53]. This is consistent with a previous study that found bee pollen to be a useful nutritional source for consumers, especially because it contains numerous antioxidant vitamins and bioactive chemicals [54].

Recent research suggests potential, but unverified, links between nutritional, metabolic, infectious, and GI factors and behavioral improvements or exacerbations of ASDs. PPA, a metabolic end product of numerous ASD-associated bacteria such as *Desulfovibrio*, *Clostridia*, and *Bacteroidetes*, has been connected to ASDs and has been shown to have extensive impacts on the intestinal permeability, brain, and behavior through the gut–brain axis [55].

One of the diseases associated with ASD patients is the “leaky gut,” or increased intestinal epithelial permeability. According to Quigley [56], 36.7% of ASD patients and their relatives (21.2%) had larger percentages of aberrant intestinal permeability than the control group (4.8%) [57]. Additionally, plasma levels of zonulin, a protein that regulates gut permeability, are higher in ASD patients; these levels appear to be correlated with the severity of the ASD symptoms [58]. The recent work by Al-Dera et al. [59] establishing the negative effects of PPA on intestinal permeability supports the reported noticeably increased levels of zonulin and occludin as indicators of gut leakiness in PPA-treated rats (Figure 2).

Additionally, Figure 2 shows that rats treated with PPA as a rodent model for ASD have significantly reduced levels of plasma zonulin and occludin, two markers of gut leakiness, after receiving a combination of probiotics and bee pollen.

According to earlier research [60,61,62], bee pollen has a variety of beneficial nutritional properties and therapeutic effects, including those that improve the gut barrier function and the immune system within the GI tract, the treatment of inflammatory status, and the prevention of oxidative stress damages [16,62,63]. The bio-accessibility of bioactive compounds from bee pollen, which is defined as the amount released during food digestion and made accessible for small intestine absorption, is what determines whether or not those compounds have beneficial effects. While numerous studies have reported the presence of beneficial nutrients in bee pollen, the outer layer of grain pollen is not easily digestible, resulting in lower nutrient bio-accessibility [64,65,66]. This could support the highly significant ameliorative effects of the synergistically used probiotics and bee pollen recorded in the current study. In the presence of probiotics, nutrient bio-accessibility of phenolics and flavonoids as active ingredients of bee pollen are much higher, probably due to pollen wall breakdown [67]. Furthermore, the release of non-extractable phenolics was probably aided by the degradation of phenolics-associated proteins and carbohydrates by the used probiotics [68]. These findings suggest that microbial compositions are crucial factors during bee pollen treatment to improve the fraction of nutrients that are available for intestinal absorption [67]. Interestingly, other research has discussed how the bacterial flora, which includes some species of lactic acid bacteria, such as *Lactobacillus kunkeii*, *Lactobacillus jensenii*, *Lactobacillus fructosus*, and *Lactiplantibacillus plantarum* (as major components of the used probiotics), influences the fermentation process [69,70]. Additionally, research indicates that in the presence of probiotics, fermentation, and spontaneous alteration enhance the nutritional and bioactive properties of bee pollen [69,71].

Table 1 demonstrates the remarkable decrease in Moraxella in PPA-treated rats fed on ND as a rodent model of autism and the remarkable increase in probiotics and bee pollen synergically protected or treated rats. This can find support in the recent work of Forsyth et al. [72] in which they recorded a 31.9% lower abundance of Moraxella in individuals with ASD compared to healthy controls, and the experimental work of Al-Dera et al. [59] in which *Moraxella* spp. was much lower in PPA-treated rats. Additional support could be found in the clinical experiment carried out by Hatakka et al. [73] which revealed a rise in *Moraxella* abundance post-treatment with probiotics.

The work of Harnett et al. [74], which finds *Candida* sp. in 33% of fecal specimens of celiac disease, a disease that clinically presents similarly to GI comorbidity in ASD patients, may also provide support for the observed increase in *Candida albicans* in PPA-treated rats. In people who are genetically prone to developing autoimmune reactions, *Candida* may act as a trigger. The fact that *Candida* is well-adapted for growth in the gut, where inflammation may alter the local bacterial ecology and result in conditions that favor *Candida* growth and inflammation, may help to explain the reported proliferation of *Candida albicans* in PPA-treated rats. The disappearance of *Candida* sp. in the probiotic and bee pollen synergistically treated rats could find support in the work of Matsubara et al. [75], which recorded that all *lactobacilli* negatively impacted *Candida albicans* yeast-to-hyphae differentiation. Thus, the *Lactobacillus* strain can be an essential tool in finding the action mechanisms of probiotics on *Candida albicans* and the prevention of candidiasis in vivo [76]. Interestingly, bee pollen may be useful for preventing *Candida* growth in the body. According to studies, bee pollen and propolis have potent anti-microbial capabilities and can help to slow the growth of a variety of *Candida* species [77,78].

## 5. Conclusions

The importance of oxidative stress increased intestinal permeability or gut leakiness indicators, and altered gut microbial diversity as etiological pathways for autism was stressed in this study. It also drew attention to the possibility of combining probiotics and bee pollen as a therapeutic intervention technique to reduce the neurotoxic effects of PPA, a short-chain fatty acid connected to the pathogenesis of autism.

## Figures and Tables

**Table 1:** Changes in bacteria growth (CFU/g) in the feces of treated rats compared to control groups.

Group	Week	*Bacilli* (Gram^+^/Gram^−^ Rod)	*Enterobacteriacea* (Gram^−^ Rod/Gram^+^ Cocci, Lactose Fermenters)	Gram^+^/Gram^−^ Rod and Cocci	*Clostridium botulinum* Gram^+^, Rod–Shaped	*Candida albicans*	*Moraxella* Spp.
Control	1	++++	+	++	−	−	++++
	2	++++	+	++	−	−	+++++
	3	++++	+	+++	−	−	+++++
	4	+++++	+	+++	−	−	+++++
PPA−Treated	1	+++	++	++	−	−	++++
	2	++++	+	++	−	−	++++
	3	++++	+	++	−	−	++
	4	+	+++	−	++	++	−
Therapeutic	1	++	++	+	++	++	++
	2	++	+	++	+	+	+++
	3	+++	++	+++	+	+	+++
	4	+++		+++	−	−	+++
Protective	1	++++	++	++	+	−	+++
	2	+++	+	++	+	−	++++
	3	++++	+	+++	−	−	++++
	4	+++	+	+++	−	−	++++

CFU: Colony-forming unit (CFU); (−) = no growth; (+) = Rare, less than 10^3^ CFU/g of feces; (++) = Few, 10^3^–10^4^ CFU/g of feces; (+++) = Moderate, 10^5^–10^6^ CFU/g of feces; (++++) = Heavy, >10^6^ CFU/g of feces.

## Data Availability

Not applicable.
